# Beyond Color Extraction: How Pulsed Electric Fields and Sulfites Affect Phenolic and Volatile Compounds of Primitivo Red Wine

**DOI:** 10.3390/foods14101792

**Published:** 2025-05-18

**Authors:** Mirella Noviello, Giusy Rita Caponio, Giuseppe Corcione, Luigi Tarricone, Rosa Anna Milella, Francesco Mazzone, Luciano Mescia, Claudio Maria Lamacchia, Fatima Zohra Makhlouf, Massimo Tripaldi, Vito Michele Paradiso, Gabriele Fioschi, Giacomo Squeo

**Affiliations:** 1 Department of Soil, Plant and Food Science, University of Bari Aldo Moro, Via Amendola 165/a, I-70126 Bari, Italy; mirella.noviello@uniba.it (M.N.); giacomo.squeo@uniba.it (G.S.); 2 Department of Bioscience, Biotechnology and Environment, University of Bari Aldo Moro, Via Orabona 4, I-70125 Bari, Italy; giusy.caponio@uniba.it; 3 Department of Biological and Environmental Sciences and Technologies, University of Salento, S.P. 6, Lecce-Monteroni, I-73100 Lecce, Italy; giuseppe.corcione@unisalento.it (G.C.); gabriele.fioschi@unisalento.it (G.F.); 4 CREA, Council for Agricultural Research and Economics, Research Centre for Viniculture and Enology, Via Casamassima 148, 70010 Turi, Italy; luigi.tarricone@crea.gov.it (L.T.); rosaanna.milella@crea.gov.it (R.A.M.); francesco.mazzone@crea.gov.it (F.M.); 5 Department of Electrical and Information Engineering, Polytechnic University of Bari, Via Orabona 4, I-70125 Bari, Italy; luciano.mescia@poliba.it; 6 IAMAtek s.r.l., Via Nicholas Green 13/A, I-70127 Bari, Italy; claudio.lamacchia@iamatek.com; 7 Higher National School of Biotechnology Taoufik Khaznadar, Nouveau Pôle Universitaire Ali Mendjeli, BP. E66, Constantine 25100, Algeria; makhlouf_f.zohra@umc.edu.dz; 8 Centro Servizi Enologici s.a.s., Via per Avetrana 57, I-74024 Manduria, Italy; enotripaldi@gmail.com

**Keywords:** pulsed electric field treatment, extraction, sulfites, red wine

## Abstract

The different impact and interaction of pulsed electric field (PEF) treatment and sulfite addition on the color, phenolic compounds, volatile profile, and sensory properties of a Primitivo wine were studied at bottling and after six months of storage. The results show that PEF treatment, promoting the electroporation of grape skin cells and the permeability of cell membranes, accelerated the extraction of anthocyanins and polyphenols only in the initial phase of maceration. After six months in bottles, wines treated only with PEF show lower levels of hydroxycinnamic acids and flavonols, but a significant increase in procyanidins B2, which are important for structure and color stability and a richer flavor profile, with higher concentrations of fruity esters and higher alcohols. The use of SO_2_ improves anthocyanin stabilization and facilitates the extraction of polyphenols. The wines from the PEF + SO_2_ combination maintain greater aromatic freshness, limiting the formation of oxidative compounds. Wines made from SO_2_ have a more balanced profile, with cherry, plum, and licorice aromas, although the combined PEF + SO_2_ treatment better preserves fresh fruit aromas, reducing the perception of dried fruits and herbaceous notes.

## 1. Introduction

The oenological sector, following new trends in wine consumption, is increasingly interested in innovative technologies to obtain higher-quality products, meet consumers’ sustainability expectations and improve production processes. In fact, innovative technologies such as ultrasound, microwave, pulsed electric fields (PEF), and high hydrostatic pressure have been developed and tested in wineries in the first steps of winemaking and during the aging process [1,2,3,4,5,6].

Among these technologies, PEF treatment is a non-thermal and environment-friendly approach tested as a novel technique to recover by-products (decreasing solvent usage, heating steps, and extraction time) [7] or in many food processing methods to preserve product or to increase the extraction rate in the food and nutraceutical industry [8,9,10,11,12]. PEF treatments induce a reversible/irreversible increment in cell membrane permeability, resulting in a process called electroporation, thanks to the application of very short electric pulses (in the range from μs to ms) at field strengths > 0.5 kV cm^−1^ to a product located between two electrodes [13].

In the oenological field, it has been proven that PEF technology accelerates the release of mannoproteins from *Saccharomyces cerevisiae* [14,15,16], and improves the malolactic fermentation performance [17,18] and wine qualities during the aging and storage period [19,20]. Moreover, when applying sufficiently strong electric fields, PEFs can exert antimicrobial activity, inactivating wine spoilage microorganisms [21,22,23], allowing a sanitization of must, and reducing the need for must sulfation [4,24,25]. Moreover, this technology has been mostly tested as new maceration trend in order to improve the extraction of phenolic and aromatic compounds from grape and reduce the maceration time and temperature [26,27].

The winemaking step known as maceration–fermentation is a crucial phase of red wine production. In this phase, the must of red grapes undergoes fermentation together with the solid parts of the grape berries, so that polyphenols and aroma compounds are extracted from the grape skin and the seeds [28]. The choice of the maceration–fermentation conditions or the different techniques used in this phase can modulate the rate of extraction and the amount of extracted phenolics and aromatic compounds [29]. When used in this winemaking step, PEF processing is applied to a mix of must, skin, and seeds obtained after destemming and light mechanical crushing. Thanks to the short pulses of high voltage and a sufficiently strong electric field, the cell membrane of grape skins undergoes electroporation, becoming permeable to compounds such as polyphenols that are otherwise unable to cross it [13]. In the last 10 years, several studies have confirmed that wines obtained from PEF-treated red grapes and macerated/fermented for a few hours or days had distinct sensory characteristics and were characterized by higher concentrations of anthocyanins, flavanols, tannins, stilbenes, hydroxycinnamic and hydroxybenzoic acids, and total polyphenols, which improve antioxidant activity, color intensity, and the formation of polymeric pigments [4,30,31,32,33]. However, in some cases, a cultivar-dependent effect of PEFs applied to maceration was reported [33,34]. López-Giral et al. (2015) reported different responses in the extraction of individual phenolic compounds in Graciano, Tempranillo, and Grenache musts [33]. López et al. (2008) also found different effects of PEF treatment at varying intensities on Graciano, Garnacha, and Mazuelo [34].

Few data are available, to the best of our knowledge, regarding the interaction of PEF treatment with must sulfiting. A previous paper evaluated their application for the microbial stabilization of white must [35]. No studies are available, instead, on the chemical effects of the interaction of PEF and sulfites in red winemaking.

The present work was therefore aimed at evaluating the different impacts and interactions of PEF treatment and sulfite addition on the color, phenolic compounds, volatile profile, and sensory properties of a Primitivo wine.

## 2. Materials and Methods

### 2.1. Grape Samples, PEF Equipment, and Processing

A batch of 400 kg of red grapes (*Vitis vinifera* var. Primitivo) was harvested in a vineyard in Manduria (Taranto, Apulia, Italy) and immediately carried out to the experimental winery of CREA (Council for Agricultural Research and Economics, Research Centre for Viniculture and Enology—Bari, Italy) for the winemaking trials.

The grapes were destemmed and crushed. Specifically, a part of the mash obtained was subjected to PEF processing in a PEF pilot plant (PULSEtek, IAMAtek s.r.l., Bari, Italy) with a chamber for batch treatments. The PEF parameters were the following: 2 kV cm^−1^ field strength, 20 μs pulse duration, 10 Hz pulse frequency, 6.5 kJ kg^−1^ total specific energy. Both PEF-treated and untreated mashes were divided into two aliquots, one of which was sulfited through the addition of 20 g hL^−1^ of potassium metabisulfite (Polsinelli s.r.l., Isola del Liri, Frosinone, Italy). Red winemaking was carried out in 25 L steel tanks. Each experimental condition was applied in triplicate.

**Figure 1:**
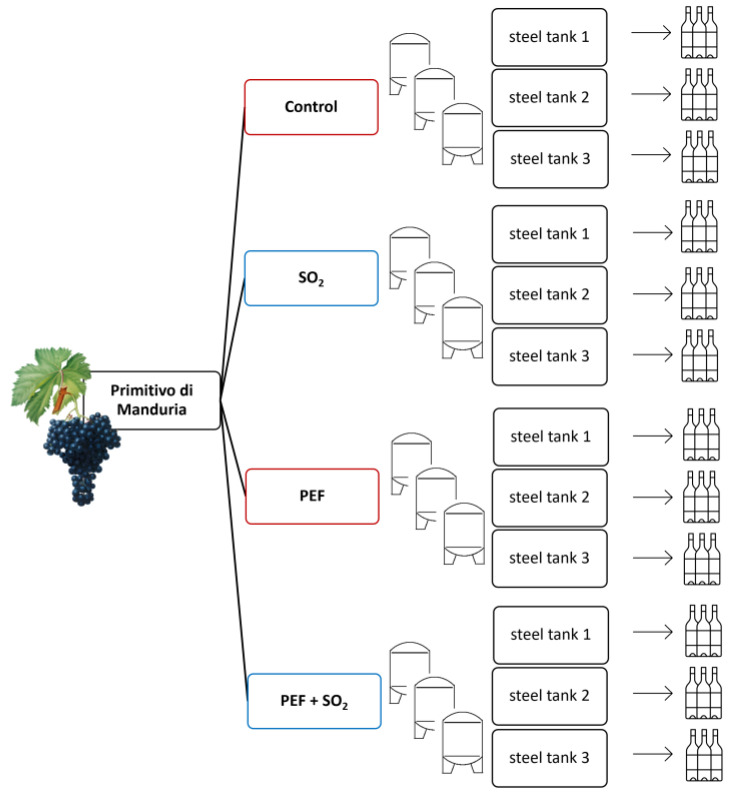
Experimental trials.

Figure 1 shows a summary of the experimental trials.

Each tank was immediately supplemented with 20 g hL^−1^ of yeast *Saccharomyces cerevisiae* (FermivinP21, OENOBRANDS SAS, Montpellier Cedex 5, France). The fermentation took place under controlled conditions at 20 °C. For all samples, maceration was performed for 15 days with 2 punch-downs per day, monitoring the color parameters (color intensity and tonality), using Agilent 8453 (Agilent Technologies, Santa Clara, CA, USA), total phenolic content, and anthocyanin content of must after one, two, five, eight, and fifteen days of maceration. Then, free-run wine was recovered by draining, and the grape pomace was pressed to recover press-run wine using an 80 L stainless steel hydro press (Enotecnica Pillan, Camisano Vicentino, Italy). The free-run and press-run wines were blended and raked after two weeks to eliminate gross lees. In order to promote the malolactic fermentation, 30 g hL^−1^ of yeast parietal extract (Enolis Scorza P, L’Enotecnica s.r.l., Nizza Monferrato, Italy), 1 g hL^−1^ of a commercial strain of *Oenococcus oeni* (Enobacter red boost, L’Enotecnica s.r.l., Nizza Monferrato, Italy), combined with 30 g hL^−1^ of a formulation composed of yeast derivatives, vitamins (B2, B5, B6) (Merck S.r.l., Milan, Italy), and potassium caseinate (Enoactiv Bacter, L’Enotecnica s.r.l., Nizza Monferrato, Italy) were added. The malic acid decrease was monitored using a Foss WineScan FT 120, as described by the manufacturer (Foss, Hillerød, Denmark). Thirty days after the conclusion of malolactic fermentation, wines were racked into dark green Bordeaux bottles. The wines obtained were analyzed at bottling and after six months of storage (18 ± 1 °C).

### 2.2. Must Characterization During Fermentation

#### 2.2.1. Determination of Total Phenolic Content, Total Anthocyanins, Color Hue, and Color Intensity

Total phenolic content (TPC) was determined using the microscale protocol [5]. Briefly, 1 mL of water, 0.02 mL of grape must, 0.2 mL of the Folin–Ciocalteu reagent (Merck S.r.l., Milan, Italy), and 0.8 mL of 10% sodium carbonate (Merck S.r.l., Milan, Italy) solution were mixed and brought to 3 mL. The absorbance was measured at 750 nm after 90 min at room temperature with a spectrophotometer Agilent 8453 (Agilent Technologies, Santa Clara, CA, USA). Results were expressed as milligrams of gallic acid equivalent per L based on a gallic acid calibration curve (50 to 500 mg L^−1^ with R^2^ = 0.998).

Total anthocyanins were determined using a pH differential protocol as previously described [36]. Appropriate grape must dilutions (1:25) were mixed with 0.025 M potassium chloride (Merck S.r.l., Milan, Italy) (pH 1) or 0.4 M sodium acetate (Merck S.r.l., Milan, Italy) (pH 4.5) buffers. Absorbance was measured at 520 and 700 nm with the Agilent 8453 spectrophotometer system (Agilent Technologies, Santa Clara, CA, USA). Results were expressed as milligram cyanidin 3-glucoside equivalents per L of grape must (mg Cy L^−1^).

The traditional method for the quantitative measurement of wine color is the Glories parameter method [37]. This method provides several parameters, the color hue (H) and the color intensity (CI), to express red wine color information through absorbance values visible at different specific wavelengths (420, 520, and 620 nm). The grape must samples were scanned and recorded using a spectrophotometer Agilent 8453 (Agilent Technologies, Santa Clara, CA, USA), with a 1 mm path length quartz cuvette. Deionized water was set as the blank reference. Each analysis was performed in triplicate.


CI = Abs420 + Abs520 + Abs620

H = Abs420/Abs520

### 2.3. Wine Characterization

#### 2.3.1. General Oenological Parameters

Ethanol (E, % *v*/*v*), titratable acidity (TA, g L^−1^), pH, malic acid (MA, g L^−1^), lactic acid (LA, g L^−1^), citric acid (CA, g L^−1^), tartaric acid (Tar, g L^−1^), ashes (g L^−1^), and free (mg L^−1^) and total SO_2_ (mg L^−1^) in all wines were analyzed in triplicate using a Foss WineScan FT 120, as described by the manufacturer (Foss, Hillerød, Denmark).

#### 2.3.2. Phenolic Composition and Color Indices

Total phenolic content (TPC as mg L^−1^ of gallic acid equivalents) was determined using the Folin–Ciocalteu reagent [38]. In a test tube, 100 µL of wine (diluted 50 times) was mixed with 100 µL of Folin–Ciocalteu reagent and, after 4 min, with 800 µL of Na_2_CO_3_ (5%). The mixture was then heated in a 40 °C water bath for 20 min, and the total phenol content was determined at 750 nm.

Total flavonoids (F, as mg L^−1^ of (+)-catechin), total anthocyanins (A, as mg L^−1^ of malvidin-3-glucoside), proanthocyanidins (Pr, as mg L^−1^ of cyanidin chloride), and flavans reactive with vanillin (FRV, as mg L^−1^ of (+)-catechin) were determined [39]. For flavonoid and anthocyanin determination, wine samples were diluted 50 times with ethanol–hydrochloric acid solution and evaluated using the absorbance spectrum in the range of 230–700 nm. The flavonoid content was calculated according to the following formula:


F = E_280_ × *df* × 82.4

E_280_ is a specific extinction coefficient at 280 nm assessed with the graphic method (absorbance corresponding to the segment, parallel to the *y*-axis, starting from the peak medium at 280 nm, and finishing on the tangent joining the points of the minimum on the left and right of the peak). *df* indicates the dilution factor, and 82.4 was the value determined considering the ratio between the concentration (mg L^−1^) and the corresponding E_280_ of pure (+)-catechin.

The anthocyanins content was evaluated using the following formula:


A = E_maximum_ × *df* × 26.6

E_maximum_ indicates the specific extinction coefficient at the maximum of the visible region (~520 nm) assessed with the graphic method as described before; 26.6 was the value determined considering the molar extinction coefficient of an anthocyanin mixture derived from grapes (Ɛ = 18800, MW medium = 500).

Absorbance at 500 nm was measured in the case of flavans reacting with vanillin. Their content in wine (diluted 5 times) was evaluated according to the following formula:


FRV = ΔE × *df*× 290.8

ΔE indicates the difference between the absorbance assessed at 500 nm (E_500_) of the sample with and without vanillin, and 290.8 was the value determined considering the ratio between the concentration (mg L^−1^) and the corresponding E500 of pure (+)-catechin.

The proanthocyanidin content was measured at 532 nm and calculated as follows:


Pr = ΔE/V × 1162.5

ΔE indicates the difference between the specific extinction coefficient assessed at 532 nm with the graphic method of the sample after and before acid hydrolysis by heat, and V is the wine volume (1 mL diluted 5 times); 1126.5 was the value determined considering the ratio between the concentration (expressed as mg L^−1^) and the corresponding ΔE of pure cyanidin chloride.

H and CI were determined using the same method described in Section 2.2.1, as well as the percentage of yellow (DO_420_(%) = DO_420_ × 100/CI), percentage of red (DO_520_(%) = DO_520_ × 100/CI), and percentage of blue (DO_620_(%) = DO_620_ × 100/CI). Moreover, the purity of the red color, dA(%), was also calculated as follows:

$$\mathrm{dA}(\%)\;\left(1-\frac{{DO}_{420}-{DO}_{620}}{2{DO}_{520}}\right)\times 100$$

Condensed tannin (Tan) was determined using the precipitation with methyl cellulose, as described by Sarneckis et al. [40].

Three experimental replicates were analyzed for each wine, using an EVOLUTION 60S UV-visible spectrophotometer (ThermoFisher Scientific, Rodano, Italy).

#### 2.3.3. Antioxidant Activity Evaluation

The DPPH (2,2-diphenyl-1-picrylhydrazyl) assay was performed on the wines according to the procedure of Tarantino et al. [41]. Briefly, each extract (50 μL) was combined with 950 μL DPPH solution (0.08 mM in ethanol) (Merck S.r.l., Milan, Italy). The decrease in absorbance was read at 517 nm using an Evolution 60S UV–visible spectrophotometer (ThermoFisher Scientific, Rodano, Italy). The results were expressed in μmol Trolox equivalents per L for all wine (μmol TE L^−1^). All determinations were carried out in triplicate. Antioxidant activity was also determined by ABTS-TEAC assay [41]. For spectrophotometry, the reaction took place directly in cuvettes by adding 50 μL of each sample to 950 μL of final ABTS + solution (Sacco S.r.l. Cadorago, Como, Italy). After 8 min, the decrease in absorbance was measured at 734 nm, using an Evolution 60S UV–visible spectrophotometer (ThermoFisher Scientific, Rodano, Italy). The results were expressed μmol Trolox equivalents per L for all wine (μmol TE L^−1^). Each sample was analyzed in triplicate.

#### 2.3.4. Analysis of Phenolic Compounds by UHPLC-DAD-MS/MS

UHPLC-DAD-MS/MS analysis of phenolic compounds was carried out using an UHPLC Ultimate 3000RS Dionex interfaced with an H-ESI II probe with an LTQ Velospro linear ion trap mass spectrometer (Thermo Fisher Scientific, Waltham, MA, USA), according to Noviello et al. [5]. Specifically, analytical separation was achieved using an Hypersil GOLD aQ C18 column (100 mm in length, 2.1 mm in internal diameter, and 1.9 µm in particle size, Thermo Fisher Scientific, Waltham, MA, USA), held at 30 °C and at a constant flow of 0.3 mL min^−1^ with a solvent A composed of water–formic acid (Merck S.r.l., Milan, Italy) (90:10 *v*/*v*) and solvent B composed of acetonitrile–formic acid (Thermo Fisher Scientific, Segrate, Milan, Italy) (99.9:0.1 *v*/*v*). The gradient program of solvent A was as follows: 0–20 min from 98% to 30%; 20–24 min isocratic at 30%. Then, equilibration was performed at the initial conditions for 9 min. The PDA detector was set to scan from 220 to 600 nm of the wavelength managed by a 3D field.

Samples were analyzed in MS with two methods: a full-scan method from 100 to 1000 m/z and a data-dependent experiment to collect MS2 data. In this case, the data-dependent settings were full-scan from 140 to 800 m/z for negative ionization mode and from 200 to 1000 for positive ionization, with an activation level of 500 counts, isolation width of 2 Da, default charge state of 2, and CID energy of 35. Tentative identification of compounds was performed using mass spectra (MS2), λmax, and retention time accordingly to the literature [42,43,44,45,46].

Analytical-grade standards were used for quantitation with the external standard method (R^2^ = 0.9972–0.9999): (+)-catechin, (−)-epicatechin, malvidin-3-O-glucoside, and quercetin were purchased from phyproof^®^ (PhytoLab, Dutendorfer, Germany); gallic acid and caftaric acid were purchased from Sigma-Aldrich (St. Louis, MO, USA). Results were expressed in mg of compound per L. All analyses were performed in triplicate.

#### 2.3.5. Analysis of Volatile Organic Compounds by SPME-GC/MS

VOCs were analyzed by SPME-GC/MS according to Prezioso et al. [47] using a Trace1300 gas chromatograph equipped with a mass spectrometer ISQ Series 3.2 SP1. One milliliter of each sample was placed into 20 mL glass vials with a headspace screw cap containing 0.2 g mL^−1^ of NaCl (to increase the ionic strength) and 10 μL of internal standard solution (2-octanol, 820 ng) (Merck S.r.l., Milan, Italy) as an internal standard for semi-quantitation, and then they closed with a silicone/PTFE septum (Merck S.r.l., Milan, Italy) and an aluminum cap. Samples were loaded into an autosampler Triplus RSH (ThermoFisher Scientific, Rodano, Italy), in which a stabilization of the headspace in the vial was obtained via equilibration for 10 min at 50 °C. The extraction was performed using a divinylbenzene/carboxen/polydimethylsiloxane (DVB/CAR/PDMS) ((Merck S.r.l., Milan, Italy) 50/30 mm SPME fiber assembly (Supelco, Bellefonte, PA, USA) at 50 °C for 30 min. The compound separation took place in a Thermo capillary column VF-WAX MS (60 m, 0.25 mm, 0.25 μm) with an injection port temperature of 200 °C and oven temperatures of 40 °C for 0.5 min, and then 3 °C min^−1^ to 210 °C with a final isothermal for 2 min. The mass detector was set as follow: ionization energy, 70 eV; detector voltage, 1700 V; scan range, 33–150 amu; source temperature, 250 °C. The tentative identification of the peaks was performed with Xcalibur v2.0 using the reference mass spectra of the NIST library. The amounts were expressed as μg of 2-octanol equivalents per liter.

#### 2.3.6. Sensory Analysis

After six months of storage, each wine was subjected to the sensory analysis following the method described by Noviello et al. [5]. The evaluation panel was composed of 7 expert tasters between 24 and 60 years of age, who knew of the ethical guidelines of the laboratory of the Food Science and Technology of the Department of Soil, Plant, and Food Science of the University of Bari (Italy). The wine samples were presented in a completely randomized order to each panelist without giving any information about their preparation. Before the first session, the descriptors to be used in the quantitative descriptive analysis (QDA) and in the list used for check-all-that-apply (CATA) analysis were defined in a preliminary consensus session with a blind simultaneous tasting of four wine samples. An evaluation sheet was provided to the judges, and the descriptors were grouped by visual (limpidity, color intensity, and viscosity), olfactory (frankness, intensity, persistence, balance), and taste (frankness, gustatory intensity, gustatory persistence, tannicity, gustatory balance, and body) characteristics; an overall judgment was also included in the evaluation sheet. Panelists rated each attribute on a scale from 0 (absence) to 10 (maximum perception). The olfactory profile was also valuated with the CATA approach: the judges reported the perception of odorous attributes (such as licorice, soft fruit, cherry, plum).

### 2.4. Statistical Analysis

Principal component analysis (PCA) was used to analyze the colorimetric index, total phenolic content, and total anthocyanins of must samples in different maceration–fermentation steps. One-way analysis of variance (one-way ANOVA), followed by Tukey’s test (*p* ≤ 0.05), was applied to determine significant differences in terms of oenological parameters between the samples. OriginPro 2024 (version 10.1.0.170 (SR0)) (OriginLab Corporation, Northampton, MA, USA) was applied for these statistical analyses. A clustering analysis with the construction of a heatmap (distance measure using Euclidean, and clustering algorithm using ward) was used to evaluate the phenolic composition, color indices, antioxidant activity, and phenolic and volatile profiles of the wine samples. Partial correlation analysis (metadata: PEF/SO_2_; covariate: SO_2_/PEF; correlation measure: point biserial correlation) was performed using Metaboanalyst 6.0. Correspondence analysis (minimum term frequency = 3) was carried out on the CATA analysis results using the KH coder software (version 3.Beta.07f) (http://khcoder.net/en/), accessed on 18 January 2025.

## 3. Results and Discussion

### 3.1. Color Trends During Maceration

**Figure 2:**
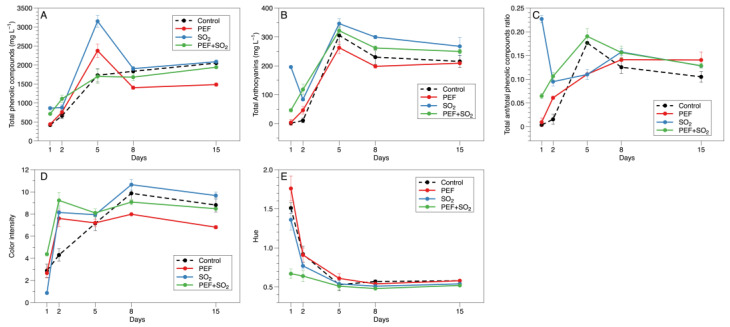
Evolution of total phenolic content (**A**), total anthocyanins (**B**), total anthocyanin/total phenolic compounds ratio (**C**), color intensity (**D**), and hue (**E**) of Primitivo musts treated and untreated with PEF and SO_2_ after one, two, five, eight, and fifteen days of maceration.

Specifically, Figure 2 shows the evolution of the total phenolic compounds, total anthocyanins, total anthocyanins/total phenolic compound ratio, color intensity, and hue during the maceration–fermentation step of the Primitivo grapes treated and untreated with PEF and SO_2_. The Supplementary Materials report the results of two-way ANOVA followed by Tukey’s HSD test (Table S1). The extraction of total phenolics (Figure 2A) showed a steep increase in the first five days, probably attributable to the hydrophilic anthocyanins, followed by a further, moderate increase in the second part of maceration, probably related to tannins [34]. Both PEF-treated and SO_2_-added musts showed a peak of total phenolic compounds after five days of maceration, with the highest values in sulfited wines. The solubilizing effect of sulfur dioxide is well known, particularly in the early stages of maceration [48,49]. Similarly, Maza et al. [27] reported that an intense PEF treatment (5 kV cm^−1^, 52.9 kJ kg^−1^) promoted a rapid release of must anthocyanins after 24 h of maceration. Moreover, the addition of sulfur dioxide determined significantly higher extraction of phenolic compounds since the first day of maceration. However, a decrease in phenolic compounds occurred in PEF-treated and SO_2_-added musts, so that the second half of maceration showed trends similar to control samples in the case of SO_2_ addition, while PEF treatment determined lower phenolic contents in macerating musts. Further research is needed to assess the fate of the lost fraction of phenolic compounds. In fact, previous research has shown that suspended cell wall materials exert a great affinity toward phenolic compounds, and that adsorption phenomena can occur and hinder the effectiveness of disruptive treatments, such as ultrasound [50,51,52,53], during maceration. This effect has been documented for ultrasound-treated grapes [52], while no evidence is available for PEF-treated grapes. Therefore, the decrease in phenolic compounds following PEF treatments remains a matter for investigation. Moreover, our findings disagree with those from previous studies on the effect of PEF treatment during red winemaking [26,27,54], which reported improved extraction performances in PEF-assisted maceration. However, several factors could explain these contrasting outcomes: a varietal effect should be considered a source of variability in the response of grapes to non-thermal technologies, for example, due to differences in the cell wall composition of grapes skins [55]; also, operational parameters and equipment engineering could largely affect the results [11]. Finally, other studies combined PEF treatment with shorter maceration times and therefore could not investigate the effects of longer maceration times [27]. The combined application of PEF and sulfur dioxide did not lead to the peak of extraction after five days, though higher levels of phenolic compounds could be found in the early stage of maceration, as observed with SO_2_ alone. Also in this case, the second half of maceration provided an extraction curve almost overlaid on the control samples. Total anthocyanins (Figure 2B) showed analogous trends. The typical extraction curve, with a peak in early–medium stages and subsequent decrease due to diminuition, could be observed in all musts. SO_2_ addition, with or without PEF, determined higher extraction in the early maceration compared to the other treatments. In the second half of maceration, only the SO_2_ treatment determined higher levels of anthocyanins compared to the control and PEF treatment. When combining SO_2_ and PEF, no significant difference could be observed with the other treatments. Therefore, in terms of extracted amounts, PEF applied to Primitivo grapes may provide improvement only for short-term macerations [56].

However, another interesting effect of the tested treatments could be observed in plotting the ratio between total anthocyanins and total phenolic compounds (Figure 2C). Though this ratio cannot be considered with quantitative purposes, due to different quantitation references for these indices, it can be informative for comparison-only purposes. The plot highlights that all the treatments, compared to the control, determined a relatively high ratio of anthocyanins to total phenols. This difference is particularly relevant in early maceration, due to the higher release of anthocyanins. However, in the final stage of maceration, significant differences could also be observed. Sulfiting and PEF + SO_2_ treatment showed, on average, higher values of this ratio compared to the control, while PEF alone gave a significantly higher ratio at the end of maceration.

As a consequence, color indices during maceration were also significantly affected by the mash treatment. The control wines showed a steep increase in color intensity (Figure 2D) up to eight days of maceration, with a subsequent slight, not significant decrease at the end of maceration. This can be considered a typical color extraction trend, reaching a maximum in initial–medium stages of maceration. All the tested treatments determined an acceleration of color extraction kinetics, with a peak of color after only two days of maceration, followed by a second peak of intensity after eight days. In particular, color intensity was on average higher when SO_2_ or PEF + SO_2_ was applied, while hue (Figure 2E) was lower, with the lowest levels found in must submitted to PEF + SO_2_ treatment. The latter case probably combined extraction enhancement with oxidative protection, allowing us to obtain optimal color indices for a Primitivo wine [57].

Therefore, besides affecting extraction kinetics, the extraction profile from Primitivo grapes was also affected by the treatments under examination.

### 3.2. Wine Characterization

#### 3.2.1. Oenological Parameters

Table 1 shows the oenological parameters of the wines at bottling and after six months of storage. In both cases, the oenological parameters evaluated showed some statistically significant differences among the theses considered, in some cases with oenological relevance. In particular, PEF treatment (with or without sulfiting) determined a significant decrease in ethanol content by around 1% vol. This could be an interesting result, considering current trends of decreasing ethanol in wines [58,59], and it suggests the opportunity to investigate the hypothesis that PEF treatment may enhance adsorption phenomena through cell wall materials, as documented for treatments with higher mechanical impact on tissues, such as ultrasounds [52,53]. On the other hand, an impact of PEF treatment on cell wall permeability, besides membrane permeability, has been well documented [60]. Evidence is documented for yeast cells, which show a strong coupling of membrane and cell wall permeability, affected by the application of PEF [60]. An analogous effect on vegetable cell walls can be postulated and should be investigated. Differences were also observed in pH and titratable acidity, though no oenological relevance could be observed [4,61]. In some studies conducted on different grape varieties, these oenological parameters were not influenced by the PEF treatment [4,27,56,62]. These results were in partial disagreement with those reported by Maza et al. [27], in which the pH, alcoholic content, and total acidity of wines (3 months after bottling) obtained from grapes of the Caladoc and Grenache varieties treated with PEF were not significantly different from control wines, also with the most intense PEF treatments.

Spontaneous malolactic fermentation started in wines during maceration, as can be observed from the data of malic and lactic acid. However, volatile acidity was still at acceptable levels, considering the pilot scale of winemaking. With regard to SO_2_, differences were observed among the wines, as expected, though the degree of difference was much lower compared to the added amounts. It should be considered that a large amount of SO_2_ is typically consumed during fermentation and bound to acetaldehyde. Therefore, the final levels of total SO_2_ were around 34 mg L^−1^ in sulfited wines and around 21 mg L^−1^ in non-sulfited wines. Correspondingly, freeSO_2_ was around 16 and 12 mg L^−1^ respectively.

The scenario after 6 months showed some relevant changes. Upon completion of malolactic fermentation, malic acid was found in traces in all wines. However, wines treated with PEF without added sulfites showed unacceptable levels of volatile acidity, indicating that PEF would have worsened the microbiological equilibrium of unprotected wines. Moreover, all wines showed a decrease in tartaric acid, related to tartaric salt precipitation. PEF-treated wines showed a more severe decrease. As the differences in ashes found among wines do not encourage us to hypothesize higher amounts of cations in PEF-treated wines, the higher precipitation rates of tartaric salts may be attributed to the higher bitartrate ion activity, as PEF-derived wines were poorer in binding counter ions such as anthocyanins [63]. Obviously, total and free SO_2_ decreased upon storage in all wines, but had similar differences compared to bottling.

#### 3.2.2. Phenolic Profile of Wines

**Figure 3:**
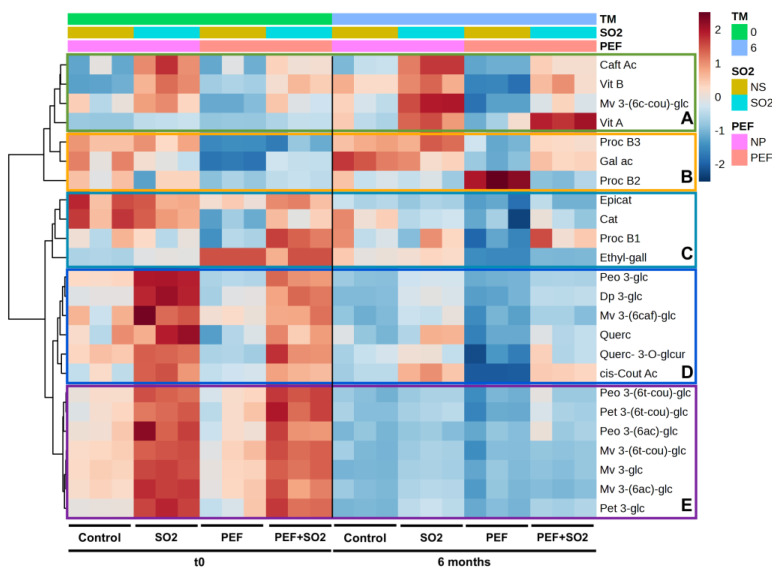
Clustering result shown as heatmap (Euclidean distance, Ward clustering algorithm) based on the phenolic profile of individual replicates of control wine, wine sample with SO_2_ (SO_2_), wine treated with PEF and SO_2_ (PEF + SO_2_), and wine treated with PEF without SO_2_ (PEF) after bottling (t0) and after 6 months. Clusters of phenolic compounds are highlighted and identified by the letters A–E. Abbreviations: Caft Ac, Caftaric Acid; Vit B, Vitisin B; Mv 3-(6c-couU-glc, Malvidin 3-(6″-t-coumaroyl)-glucoside; Vit A, Vitisin A; Proc B3, Procyanidin B3; Gal ac, Gallic acid; Proc B2, Procyanidin B2; Epicat, (-)-epicatechin; Cat, (+)-catechin; Proc B1, Procyanidin B1; Ethyl-gall, Ethylgallate; Peo 3-glc, Peonidin 3-(6″-t-coumaroyl)-glucoside; Dp 3-glc, Delphinidin 3-glucoside; Mv 3-(6caf)-glc, Malvidin 3-(6″-caffeoyl)-; Querc, Quercetin; Quer-3-O-glcur, Quercetin- 3-O-glucuronide; cis-Cout Ac, *cis*-Coutaric acid; Peo 3-(6t-cou)-glu, Peonidin 3-(6″-t-coumaroyl)-glucoside; Pet 3-(6t-cou)-glc, Petunidin 3-(6″-t-coumaroyl)-glucoside; Peo 3-(6ac)-glu, Peonidin-3-(6″-acetyl)-glucoside; Mv 3-(6t-cou)-glc, Malvidin 3-(6″-t-coumaroyl)-glucoside; Mv 3-glc, Malvidin 3-glucoside; Mv 3-(6ac)-glc, Malvidin 3-(6″-acetyl)-glucoside; Pet 3-glc, Petunidin 3-glucoside.

Multivariate analysis was applied to the phenolic profiles of wines to better evaluate the influence of the interaction of PEF treatment with the use of SO_2_ [47,64]. The raw data are shown in Supplementary Table S2. Figure 3 shows the heatmap resulting from cluster analysis (Euclidean distance, Ward’s clustering algorithm) performed on the phenolic profile of individual replicates of control wine, wine treated with SO_2_, wine treated with only PEF, and wine treated with PEF and SO_2_, analyzed after bottling (t0) and 6 months after bottling. The color mapping corresponds to the relative concentration of the auto-scaled data, with the samples in the columns and the metabolites in the rows. The phenolic compounds were grouped into five main clusters, indicated with the letters A, B, C, D, and E.

Group A, including Vitisins A and B, caftaric acid, and acylated malvidin, clustered compounds were found in higher amounts in wines treated with SO_2_. Vitisins A and B are condensation pyrananthocyanins involving pyruvic acid and acetaldehyde, respectively [65], and are much more stable than the original anthocyanins helping to maintain the wine’s deep red color over time and reducing the risk of discoloration turning to brown or orange while maintaining garnet and purplish hues. Their presence indicates good fermentation and proper maturation [66,67]. The presence of sulfur dioxide probably prevented malolactic fermentation while maintaining a good presence of the precursors that in six months allowed for the formation of these condensation products [68]. This is also confirmed by the low levels of lactic acid in the theses with SO_2_ compared to those in the control theses (Table 1).

Bringing attention to clusters B and C, which group mainly flavanols (catechins and procyanidins), the increase in procyanidin B2 in the thesis using only PEF without sulfur dioxide six months after bottling is quite evident, bringing the value of this compound from 57.5 mg L^−1^ at time 0 to 88.5 mg L^−1^. This polyphenolic compound is a dimer of catechins (it consists of two molecules of (-)-epicatechin) and plays a key role in building the body and structure of wine, making it more robust. In addition, procyanidin B can interact with anthocyanins to form stable pigment complexes (this phenomenon contributes to color stability in aged red wines) and helps protect wine from oxidation, prolonging its longevity and preserving its organoleptic characteristics [69,70,71]. Procyanidin B1 is also more represented in the trials with PEF together with SO_2_ both in the analyses at time 0 (49.1 mg L^−1^) and in those carried out six months after bottling (44.9 mg L^−1^), while it is much less so with the use of PEF alone (35.5 mg L^−1^ and 25.2 mg L^−1^, respectively, at time 0 and after 6 months). Catechins, on the other hand, are mostly present in analyses performed at time 0 in descending order in the control (48.6 mg L^−1^ and 31.8 mg L^−1^ for catechin and epicatechin, respectively), trials with SO_2_ alone (46.2 mg L^−1^ and 29.5 mg L^−1^ for catechin and epicatechin, respectively), trials with PEF and SO_2_ together (40.7 mg L^−1^ and 29.0 mg L^−1^ for catechin and epicatechin, respectively), and trials with PEF alone (30.7 mg L^−1^ and 24.1 mg L^−1^ for catechin and epicatechin, respectively). Procyanidin B3 was more represented in the theses where there was no use of PEF (67.7 mg L^−1^ and 67.9 mg L^−1^ in control and SO_2_ theses, respectively, compared to 47.7 mg L^−1^ and 50.3 mg L^−1^ in PEF and PEF + SO_2_ theses, respectively). At zero time, however, greater presence of ethyl-gallate is shown with the use of PEF alone and with use of PEF and SO_2_. Therefore, wines obtained with PEF technology showed a phenolic profile characterized by lower levels of catechins.

As regards clusters D and E, which include mainly anthocyanins and flavonols, the heatmap showed a decrease after six months of maturation compared to time 0 for almost all the compounds, except for *cis*-coutaric acid and quercetin, which were more represented after six months in the theses with SO_2_ both with and without PEF. In the area of the time 0 heatmap of groups D and E, higher levels of anthocyanins and flavonols were determined through the use of sulfur dioxide, both with and without PEF. The addition of SO_2_ at the beginning of maceration increased, as expected, the concentration of individual anthocyanins, due to increased extractability and antioxidant protection [63], and oxidizable catechins. It is known that the addition of SO_2_ at crushing results in the transfer of polyphenols from the skins to the must, and their antioxidant effect changes the color structure of the final wine [72,73]. The wine obtained with the PEF treatment alone was similar to the control wine without SO_2_ in terms of the profile of phenolic compounds, although lower levels of flavonols (free and glycosylated quercetin), flavanols (catechin, procyanidin B1 and procyanidin B3), and peonidin 3-glucoside and delphinidin 3-glucoside were observed both at time 0 and after six months of bottling.

In addition, analyses showed low hydroxycinnamic acid levels (caftaric and cutaric acid) in trials using only PEF without sulfur dioxide, both immediately after fermentation and six months after bottling. These results are in disagreement with what has been reported in the literature where this technology has been applied to grape marc and grape skins during the early stages of winemaking to improve final wine quality or reduce maceration time [6]. In all these cases, PEF treatments resulted in a wine with a higher amount of phenolic compounds, but considering shorter maceration times (mainly in the range of 1–6 days) than those used in our study [27,33,74]. Therefore, this study highlights the need to investigate the fate of phenolic compounds when longer maceration times are adopted with PEF-treated grapes, since the increase in cell membrane and wall permeability caused by electroporation may induce a release of phenolic compounds during the first few days of maceration (see Figure 2), but subsequent phenomena occurring during prolonged maceration could lead to the loss of the advantage gained in the early phases [75,76]. The change in cell wall permeability induced by PEF in crushed grapes would contribute to also explaining the varietal response that was previously reported regarding the effect of PEF in red winemaking and that could also be hypothesized to explain the present results [26,31,77]. Primitivo, in particular, is well known for its thin skin [78,79]. Therefore, the effect derived from membrane and cell wall permeabilization induced by PEF to Primitivo grapes could have not been higher than that derived from the solubilizing activity of SO_2_.

#### 3.2.3. Volatile Profile

**Figure 4:**
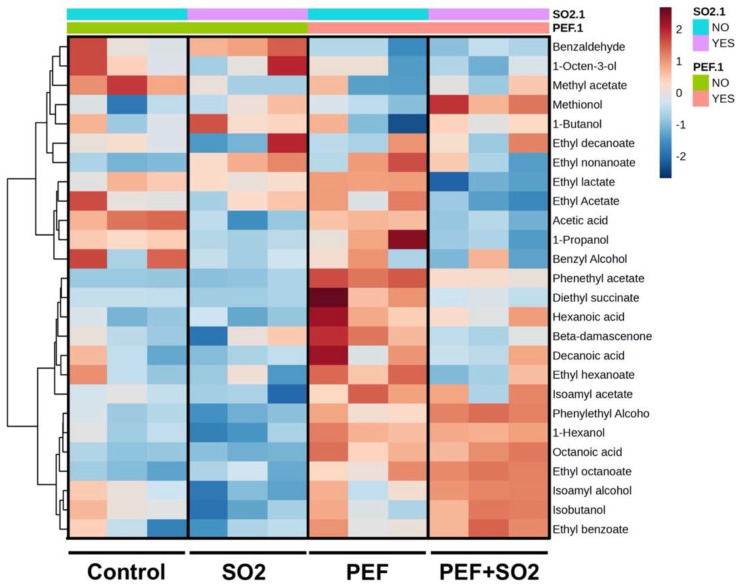
Clustering result shown as a heatmap (Euclidean distance, Ward clustering algorithm) based on the volatile profile of individual replicates of control wine, wine sample with SO_2_ (SO_2_), wine treated with PEF and SO_2_ (PEF + SO_2_), and wine treated with PEF without SO_2_ (PEF) after 6 months.

Figure 4 exhibits the clustering result shown as a heatmap (Euclidean distance, Ward clustering algorithm) based on the volatile profile of individual replicates (A,B,C) of control wine, wine sample with SO_2_ (SO_2_), wine treated with PEF and SO_2_ (PEF + SO_2_), and wine treated with PEF without SO_2_ (PEF) after 6 months. The heatmap clearly shows that after six months of storage the wine samples presented clearly different profiles, influenced by both PEF treatment and sulfitation.

The control wines showed profiles characterized by two clusters of compounds, mainly including fusel alcohols and some ethyl esters. A few compounds found in these clusters could be related to defects, of both microbiological and oxidative origin (1-octen-3-ol, acetic acid, ethyl acetate, benzaldehyde), or indicated enhanced bacterial activity (ethyl lactate). The use of SO_2_ did not substantially determine changes in the volatile profile of the wines, even though the low level of acetic acid could be related to the microbiological protection of sulfur dioxide. The wines from grapes that had undergone PEF treatment showed richer volatile profiles, compared to control wine and sulfited wine. PEF wines presented higher levels of the cluster of volatiles including acetic acid, ethyl acetate, and ethyl lactate, indicating a microbial complexity comparable to the control wine. On the other hand, PEF wines showed higher levels of β-damascenone (a norisoprenoid that enhances fruity notes) [80], isoamyl acetate, and ethyl esters of organic acids such as ethyl hexanoate and ethyl octanoate with fruity notes derived from yeast activity during fermentation [81], as well as 2-phenyl ethanol (a higher alcohol with notes of rose). β -damascenone, in particular, has been shown to be a molecule that amplifies the fruity notes of wines in addition to imparting its typical baked apple [80]. In addition to β-damascenone, isoamyl alcohol is also a compound that plays an important role in the olfactory profile of wine. Both amplify the fruity notes and their intensity [82]. Relevant levels of other acyl and ethyl esters such as phenyl-ethyl acetate (honey notes) and diethyl succinate could be observed. The presence of ethyl esters in PEF treatment samples may suggest that the treatment may have stimulated the esters’ production by the yeasts. Those compounds tend to diminish during aging due to hydrolysis reactions [83]: PEF-treated samples presented higher amounts than untreated ones after 6 months. Instead, the presence of 1-propanol, normally produced by yeasts due to complications in the uptake of nitrogen sources during fermentation [84], remains to be further investigated. The latter, along with acetic acid is also present in the control sample. Presumably, these may be products derived from the oxidative metabolism of indigenous yeasts and other microorganisms that sulfur dioxide is normally capable of limiting [85]. In addition, methylacetate was found at higher levels in the control samples, while control and SO_2_-only samples were characterized by the presence of 1-octen 3-ol, with the typical earthy mushroom odor that is usually produced by some fermenting yeasts and fungi. This compound results from the fatty acid metabolism of this microorganism that we normally find on the grape skin [86]. The PEF treatment combined with the addition of SO_2_ shares the presence of higher concentrations of ethyl-octanoate, 2-phenylethanol, and isoamyl acetate with the PEF-only treatment. Based on the results of this study, PEF treatment combined with reduced doses of SO_2_ could be evaluated in order to pursue the goal of optimized sensory properties along with sulfur dioside reduction, in accordance with the study of Lisanti et al. [85], which concluded that, currently, no single alternative matches the efficacy of SO_2_, which provides both antimicrobial protection and antioxidant action. This study shows that the extraction and preservation of noble compounds needs the combination of those alternative methods with the action of sulfur dioxide. The best approach could be, therefore, to reduce SO_2_ levels through a combined strategy with complementary methods.

**Figure 5:**
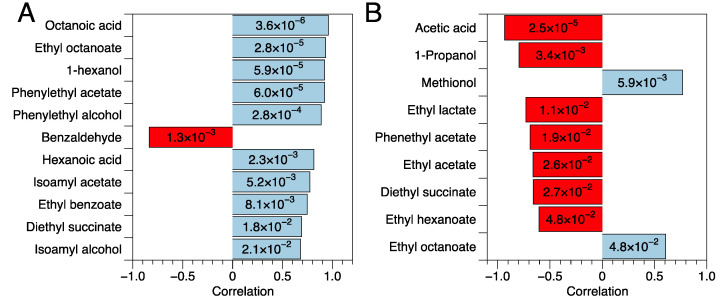
Results of point biserial partial correlations of chemical parameters with the PEF treatment ((**A**), levels: wine with PEF, wine without PEF) and the use of SO_2_ ((**B**), levels: wine with SO_2_, wine without SO_2_). SO_2_ and PEF were considered covariates of interest for A and B, respectively. Only correlations with *p*-value < 0.05 (reported in labels) were included.

Moreover, Figure 5 reports the results of point biserial partial correlation analysis of the volatile data with the PEF treatment variable (levels: wine with PEF, wine without PEF) and SO_2_ variable (levels: wine with SO_2_, wine without SO_2_) for the wines analyzed six months after bottling. The PEF treatment was positively correlated with some esters (i.e., ethyl octanoate, phenylethyl acetate, or isoamyl acetate), carboxylic acids (i.e., octanoic acid, hexanoic acid), higher alcohols (i.e., 1-hexanol, isoamyl alcohol, phenylethanol), and β-Damascenone. The positive correlation between the PEF treatment and most of the esters found in the wines (Figure 5) may suggest that the treatment may have stimulated the ester production of the yeasts, compounds that tend to diminish during aging due to hydrolysis reactions [83]. On the other hand, the PEF treatment was negatively correlated with benzaldehyde. Despite the functional group, a positive correlation emerged regarding some fermentation-related carbon backbones (C6–C8 straight chain, isoamyl chain, phenylethyl group): this suggests that the impact of PEF on the volatile profile may be due to an effect on yeast metabolism. A possible increase in oxidative stability should be investigated, as suggested by the negative correlation of PEF treatment with benzaldehyde.

Differently, the use of SO_2_ was positively correlated with methionol (related to the reductive environment) and ethyl octanoate. On the contrary, SO_2_ was negatively correlated with volatile related to bacterial activity (acetic acid, ethyl acetate, ethyl lactate), 1-propanol, and a few esters (i.e., phenyethyl acetate, diethyl succinate, ethyl hexanoate). The effect of SO_2_ on the synthesis of esters, mainly medium-chain fatty acid (MCFA) esters, has been reported previously [87,88,89,90], though the outcomes are not univocal, and both increases and decreases in MCFA esters have been observed. The hypothesized mechanisms underlying this effect are related to oxygen availability and fatty acid metabolism.

#### 3.2.4. Sensory Analysis

**Figure 6:**
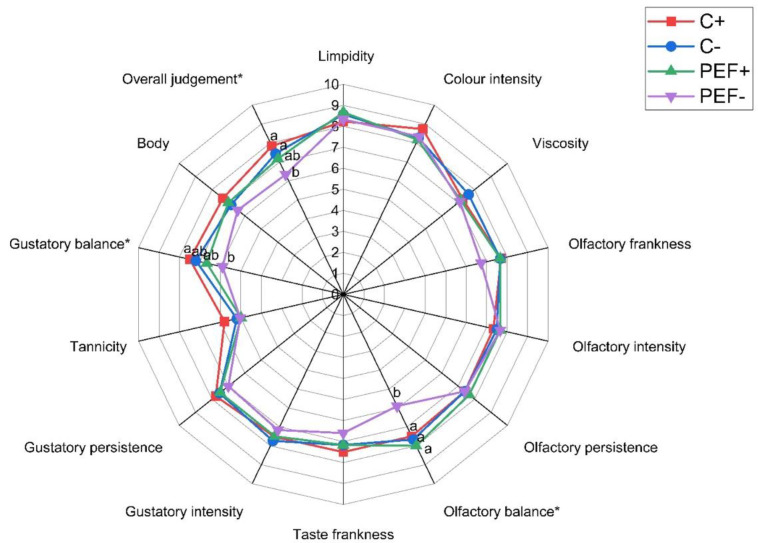
Results of sensory analysis and overall judgment of wines during storage. * Sensory parameters where there is a significant difference among the wines (Tukey test, *p <* 0.05). Abbreviations: C+, control sample with SO_2_; C−, control sample without SO_2_; PEF+, sample treated with PEF and SO_2_, PEF−, sample treated with PEF without SO_2_.

The results of the sensory analysis of the quality parameters of wines after six months of storage are reported in Figure 6. The analysis of variance showed that no significant differences were found between the samples for most of the parameters considered, and the sensorial quality of the wines was almost comparable. Differences in color intensity were not perceived by the judges. However, the analysis of variance demonstrated statistically significant differences for three of the descriptors considered: olfactory and gustatory balance and overall judgment. The PEF-treated wine received the lowest score from the judges, while the other wines were comparable.

**Figure 7:**
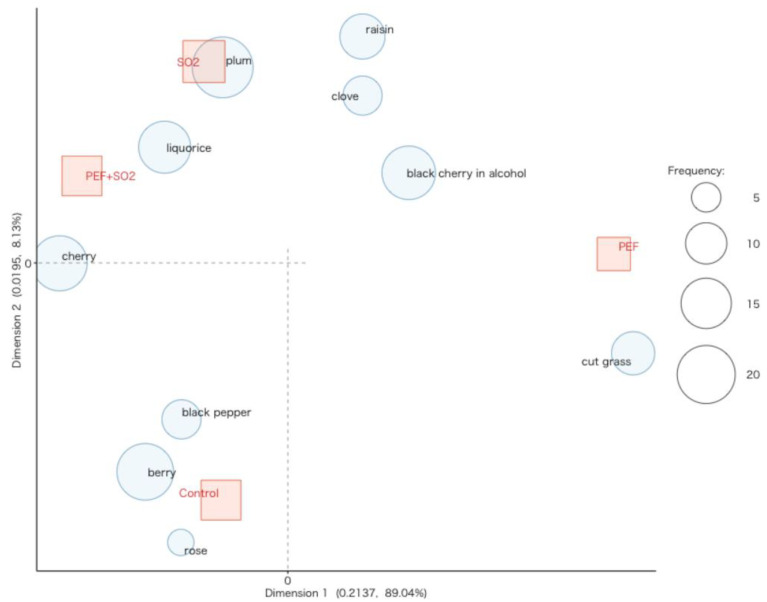
Correspondence analysis carried out on the results of the CATA analysis. Aroma descriptors perceived by the judges in control wine (Control); control wine with SO_2_ (SO_2_); wine treated with PEF (PEF), and wine treated with PEF and SO_2_ (PEF + SO_2_) after six months of storage.

Figure 7 shows the results of the correspondence analysis applied to the citation frequencies of the aroma descriptors that the judges identified in each wine by smell during CATA analysis. The table of frequencies is reported as Table S3 in the Supplementary Materials. The largest part of variability, along dimension 1, was due to PEF wine, distinguished from the other wines by herbaceous notes described as *cut grass*. This may be the reason for the lower quality attributed to this wine by the panelists. Control wine is positioned in the lower-left quadrant, characterized by rose, berry, and black pepper descriptors. The upper-left quadrant includes wines with added SO_2_, whose characterizing descriptors referred to fresh fruit descriptors (cherry), processed fruit (black cherry in alcohol, plum, raisins), clove, and licorice. Interestingly, the combined use of PEF and SO_2_ was effective in keeping fresh fruit scents with respect to dried fruit scents (plum, raisins), which could not always be appreciated by consumers, being also associated with premature oxidation defects [91].

## 4. Conclusions

This study shows how the application of pulsed electric fields (PEFs) to Primitivo crushed grapes determined effects that go beyond the expected increase in extracted phenolic compounds and can interact with the use of SO_2_. Unexpected outcomes, in fact, were observed during two-week maceration. The results suggest that PEF treatment, promoting the electroporation of grape skin cells by increasing the permeability of cell membranes, accelerated the extraction of anthocyanins and polyphenols during the first few days of maceration, although we thereafter observed a loss in the early gain in extracted phenolic compounds, whose fate should be investigated to assess either reabsorption or degradation. The behavior observed in this study might be partially attributed to variety-dependent variables, as grape cultivars differ in skin thickness, cell wall composition, and phenolic profiles, all of which can influence the response to electroporation. It is worth noting that these observations were made on Primitivo grapes, and further investigations on different varieties could reveal different dynamics of extraction, stabilization, and reabsorption processes. However, the length of the maceration step could be a critical variable requiring optimization. The addition of SO_2_ compensates for these limitations by stabilizing the phenolic compounds and preventing their oxidation, thus ensuring greater overall process efficiency.

Further, treatment with PEF (both with and without SO_2_) resulted in a decrease in ethanol content of about 1% vol, a significant result in line with the wine consumer’s trend toward lower alcohol content wines. This may also be the result of ethyl alcohol reabsorption by the skins during maceration, along with the other compounds. After six months in bottles, wines obtained using the treatment with PEF and without SO_2_ showed high levels of volatile acidity, indicating microbiological problems in the absence of sulphites. The phenolic profile of the samples shows that wines treated only with PEF show lower levels of hydroxycinnamic acids and flavonols, but a significant increase in procyanidin B2, which are important for structure and color stability. Control wines show a stable phenolic profile, with flavanols well represented. The use of SO_2_ improved anthocyanin stabilization. The PEF + SO_2_ combination provides a balance between the extraction and stabilization of phenolic compounds. The volatile profile of the samples tested showed that PEF-treated wines had a richer flavor profile, with higher concentrations of fruity esters and higher alcohols. The wines from the PEF + SO_2_ combination, on the other hand, maintain greater aromatic freshness, limiting the formation of oxidative compounds. Wines made using SO_2_ alone or PEF + SO_2_ have a more balanced profile, with cherry, plum, and licorice aromas, although the combined PEF + SO_2_ treatment better preserves fresh fruit aromas, reducing the perception of dried fruits and herbaceous notes.

## Figures and Tables

**Table 1:** Oenological parameters of Primitivo di Manduria wine at bottling and after six months of storage ^a^.

	At Bottling				6 Months			
	Control	SO_2_	PEF + SO_2_	PEF	Control	SO_2_	PEF + SO_2_	PEF
E (% *v*/*v*) ^b^	16.51 ± 0.29 a	16.7 ± 0.1 a	15.77 ± 0.02 b	15.38 ± 0.11 b	16.53 ± 0.37 a	16.88 ± 0.08 a	15.87 ± 0.02 b	15.51 ± 0.10 b
VA (g L^−1^)	0.51 ± 0.04 bc	0.44 ± 0.01 a b	0.41 ± 0.00 c	0.56 ± 0.05 a	0.67 ± 0.02 a	0.51 ± 0.02 a	0.55 ± 0.08 a	1.02 ± 0.41 a
TA (g L^−1^)	4.95 ± 0.12 a b	4.97 ± 0.05 a b	5.12 ± 0.03 a	4.81 ± 0.06 b	4.69 ± 0.01 a	4.77 ± 0.02 a	4.73 ± 0.11 a	5.46 ± 0.84 a
pH	3.87+0.01 a	3.86 ± 0.01 a b	3.80 ± 0.01 c	3.84 ± 0.01 b	3.89 ± 0.02 a	3.84 ± 0.02 a b	3.79 ± 0.02 b	3.78 ± 0.06 b
MA (g L^−1^)	0.12 ± 0.02 bc	0.19 ± 0.04 a b	0.23 ± 0.03 a	0.06 ± 0.01 c	0.02 ± 0.02 a	0.07 ± 0.01 a	0.05 ± 0.05 a	0.04 ± 0.02 a
LacA (g L^−1^)	0.69 ± 0.01 a	0.66 ± 0.03 a	0.51 ± 0.02 b	0.71 ± 0.09 a	0.86 ± 0.03 a b	0.67 ± 0.03 b	0.69 ± 0.07 b	1.59 ± 0.67 a
TarA (g L^−1^)	2.41 ± 0.01 b	2.59 ± 0.01 a	2.32 ± 0.01 b	2.31 ± 0.09 b	2.06 ± 0.07 a b	2.22 ± 0.08 a	1.82 ± 0.04 c	1.84 ± 0.14 bc
CitA (g L^−1^)	0.14 ± 0.01 b	0.15 ± 0.01 b	0.17 ± 0.01 a	0.13 ± 0.01 b	0.09 ± 0.01 b	0.13 ± 0.01 a b	0.14 ± 0.02 a	0.13 ± 0.02 a b
Ashes (g L^−1^)	3.11 ± 0.06 b	3.30 ± 0.01 a	3.04 ± 0.03 bc	3.01 ± 0.02 c	3.12 ± 0.10 a	3.18 ± 0.09 a	2.85 ± 0.02 a b	2.61 ± 0.28 b
Free SO_2_ (mg L^−1^)	12.0 ± 1.0 b	16.5 ± 0.5 a	15.3 ± 0.6 a	11.7 ± 0.6 b	9.5 ± 0.7 b	13.7 ± 1.2 a	12.0 ± 1.0 a	8.7 ± 0.6 b
Total SO_2_ (mg L^−1^)	21.0 ± 1.0 b	34.5 ± 0.5 a	34.3 ± 2.3 a	20.7 ± 0.6 b	16.0 ± 3.0 b	26.3 ± 2.5 a	24.0 ± 1.7 a	16.3 ± 1.5 b
TPC (mg L^−1^ gallic acid)	2774.1 ± 70.8 a	2777.7 ± 67.9 a	2504.1 ± 46.7 b	1970.8 ± 30.2 c	2559.1 ± 26.4 a	2932.2 ± 242.4 a	2522.2 ± 111.8 a	1941.9 ± 55.5 b
DPPH (µmol troloxeq L^−1^)	9863.1 ± 111.4 a	10,103.0 ± 104.5 a	8368.2 ± 51.6 b	6499.5 ± 272.6 c	8979.3 ± 283.6 a	9037.4 ± 69.6 a	9110.6 ± 309.1 a	7714.1 ± 721.1 a
ABTS (µmol troloxeq L^−1^)	11,488.9 ± 375.4 a	11,627.8 ± 677.9 a	10,946.3 ± 373.4 a	8725.9 ± 375.2 b	11,975.9 ± 74.9 a	11,731.5 ± 86.1 a	11,998.1 ± 396.5 a	8742.6 ± 456.8 b
A (mg L^−1^)	360.2 ± 51.1 b	445.5 ± 49.4 a b	457.5 ± 19.4 a	300.5 ± 6.7 c	226.07 ± 11.1 b	297.4 ± 4.0 a	260.2 ± 32.4 a b	188.5 ± 6.8 c
F (mg L^−1^ (+)-Catechin)	1866.5 ± 84.5 a	1870 ± 150.8 a	1664.2 ± 74.1 a	1339.6 ± 53.9 b	1646.6 ± 51.5 a	1681.3 ± 17.2 a	1433.7 ± 58.0 b	1114.0 ± 38.37 c
Pr (mg L^−1^ Cyanidin Chloride)	1470.9 ± 142.7 a	15,519 ± 38.2 a	1481.1 ± 166.0 a	1119.3 ± 40.8 b	1664.0 ± 62 a	1783.4 ± 162.1 a	1641.7 ± 80.5 a	1182.0 ± 38.3 b
Tan (mg L^−1^ Epicatechin)	1569.6 ± 101.4 b	1538.7 ± 23.8 b	1847.9 ± 155.2 a	1099.2 ± 29.1 c	1625.8 ± 90.3 a	1618.7 ± 142.5 a	1292.5 ± 56.9 b	984.6 ± 67.4 c
FRV (mg L^−1^ (+)-Catechin)	764.9 ± 80.0 a	628.9 ± 52.3 a b	496.1 ± 593.4 a b	498.2 ± 75.7 b	620.5 ± 39.7 a	667.3 ± 31.0 a	373.7 ± 397.6 b	364.6 ± 96.3 b
FRV/Pr	0.52 ± 0.04 a	0.41 ± 0.03 a	0.40 ± 0.08 a	0.44 ± 0.06 a	0.37 ± 0.03 a b	0.38 ± 0.02 a	0.24 ± 0.02 b	0.31 ± 0.09 a b
CI	0.92 ± 0.03 b	1.08 ± 0.02 a	0.89 ± 0.04 b	0.67 ± 0.03 c	0.91 ± 0.04 b	1.12 ± 0.05 a	0.90 ± 0.07 b	0.68 ± 0.02 c
T	0.69 ± 0.01 a	0.63 ± 0.01 b	0.64 ± 0.01 b	0.71 ± 0.01 a	0.74 ± 0.01 a	0.66 ± 0.01 b	0.67 ± 0.01 b	0.72 ± 0.01 a
DO_420_ (%)	35.64 ± 0.06 b	33.75 ± 0.39 b	34.01 ± 0.18 a	35.97 ± 0.45 a	37.07 ± 0.05 a	34.41 ± 0.28 b	35.09 ± 0.38 b	36.64 ± 0.58 a
DO_520_ (%)	68.92 ± 1.37 b	90.61 ± 4.12 a	73.47 ± 2.41 b	48.48 ± 2.02 c	61.47 ± 2.61 b	88.48 ± 5.89 a	69.60 ± 7.32 b	47.16 ± 1.86 c
DO_620_ (%)	0.33 ± 0.02 b	0.42 ± 0.01 a	0.34 ± 0.03 b	0.24 ± 0.02 c	0.31 ± 0.02 b	0.43 ± 0.02 a	0.33 ± 0.03 b	0.24 ± 0.02 c
dA (%)	77.83 ± 0.09 b	80.53 ± 0.44 a	80.15 ± 0.23 a	77.35 ± 0.81 b	75.78 ± 0.09 b	79.86 ± 0.36 a	78.61 ± 0.49 a	76.42 ± 1.17 b

^a^ Average ± standard deviation (*n* = 3). In the rows and for each time considered, different letters following data indicate statistically significant differences at *p* < 0.05 according to one-way ANOVA followed by Tukey’s test. ^b^ Abbreviations: SO_2_, wine sample with SO_2_; PEF + SO_2_, sample treated with PEF and SO_2_; PEF, sample treated with PEF without SO_2_; E, ethanol; VA, volatile acidity; TA, titratable acidity; pH; MA, malic acid; LacA, lactic acid; TarA, tartaric acid; CitA, citric acid; TPC, total phenolic content; DPPH and ABTS antioxidant activity assays; A, total anthocyanins; F, total flavonols; Pr, proanthocyanins; Tan, condensed tannins; FRV, flavans reactive with vanillin; CI, color intensity; T, tonality; DO_420_ (%), DO_520_ (%), DO_620_ (%), percentage of yellow, red, and blue color; dA (%), purity of red color.

## Data Availability

The original data presented in the study are openly available from FigShare at DOI: https://doi.org/10.6084/m9.figshare.28765742.v1.

## Supplementary Materials

The following Supporting Information can be downloaded at https://www.mdpi.com/article/10.3390/foods14101792/s1: Table S1: Results of two-way ANOVA and post hoc Tukey’s test for multiple comparisons related to the parameters reported in Figure 2; Table S2: Raw data of the analysis of phenolic compounds; Table S3: Frequency of descriptors reported by the panelists.
