# Supplementary material for: Beyond Color Extraction: How Pulsed Electric Fields and Sulfites Affect Phenolic and Volatile Compounds of Primitivo Red Wine
Source: Foods. 2025 May 18;14(10):1792. doi: 10.3390/foods14101792 (PMC12110841; doi:10.3390/foods14101792)
Supplement: Supplementary file 1 [file foods-14-01792-s001.zip › foods-3605339-supplementary.pdf]

Table S1. Results of two-way ANOVA and post-hoc Tukey's test for multiple comparisons related to the parameters reported in Figure 2.[a]

		Total phenolic compounds (TPC)	Total anthocyanins (T-Ant)	T-Ant/TPC ratio	Color intensity	Hue
Treatment		p = 4.9234e-16 ***	p = 5.1363e-19 ***	p = 8.4790e-19 ***	p = 1.6386e-10 ***	p = 3.9151e-14 ***
C		bc	c	c	b	a
PEF		c	c	c	b	a
S		a	a	a	a	b
PEF+S		b	b	b	a	c
Days		p = 3.0499e-32 ***	p = 2.9758e-32 ***	p = 9.9447e-22 ***	p = 1.3390e-28 ***	p = 4.0529e-26 ***
	1	e	c	c	d	a
	2	d	c	c	c	b
	5	a	a	a	c	c
	8	c	b	a	a	c
	12	b	b	b	b	c
Interaction		p = 3.9608e-16 ***	p = 8.4533e-11 ***	p = 1.2635e-22 ***	p = 1.1096e-13 ***	p = 1.3221e-13 ***
	1	j	j	i	gh	b
	2	ij	j	i	g	c
C	5	def	abc	bc	ef	e
	8	cde	defg	efg	ab	de
	15	cd	efg	fg	bcd	de
	1	j	j	i	h	a
	2	i	ij	h	def	c
PEF	5	b	cde	fg	ef	de
	8	g	g	de	cdef	e
	15	fg	fg	de	f	de
	1	hi	g	a	i	b
	2	hi	hi	g	cdef	cd
S	5	a	a	fg	cdef	e
	8	cde	bc	cd	a	e
	15	c	cd	ef	ab	e
	1	ij	ij	h	g	de
	2	h	h	fg	bc	de
S+PEF	5	ef	ab	b	cdef	e
	8	efg	cde	cde	bc	e
	15	cde	def	ef	bcde	E

[a], different letters mean significant difference at $p < 0.05$, with *a* indicating the highest mean in the comparison.

Table S2. Raw data of the analysis of phenolic compounds

Thesis	SO_2	PEF	Time (Months)	Delphinidin 3-glucoside	Petunidin 3-glucoside	Peonidin 3-glucoside	Malvidin 3-glucoside	Vitisin A	Vitisin B	Peonidin-3-(6''-acetyl)-glucoside	Malvidin 3-(6''-acetyl)-glucoside	Malvidin 3-(6''-caffeoyl)-glucoside	Petunidin 3-(6''-t-coumaroyl)-glucoside	Malvidin 3-(6''-c-coumaroyl)-glucoside	Peonidin 3-(6''-t-coumaroyl)-glucoside	Malvidin 3-(6''-t-coumaroyl)-glucoside	Gallic acid	Caftaric Acid	*cis*-Coutaric acid	Ethylgallate	Procyanidin B1	(+)-catechin	Procyanidin B2	Procyanidin B3	(-)-epicatechin	Quercetin- 3-O-glucuronide	Quercetin
Control	NO	NO	0	10.0	27.9	27.4	648.0	8.5	2.7	3.0	53.1	5.1	8.3	2.7	7.4	58.4	189.6	44.6	7.4	18.7	41.3	51.3	82.7	70.2	35.6	1.2	0.8
Control	NO	NO	0	10.3	28.6	26.9	688.6	8.3	2.7	3.0	56.7	3.6	9.6	2.0	8.2	60.3	161.9	64.0	10.2	19.2	37.5	42.9	70.5	66.5	26.3	1.3	0.6
Control	NO	NO	0	10.3	29.0	28.0	666.1	8.3	3.0	3.5	54.3	5.2	10.3	2.5	8.1	62.9	189.0	45.5	7.3	18.6	44.8	51.5	83.0	66.4	33.5	1.2	1.1
Control	NO	NO	6	4.5	13.4	14.7	324.2	17.3	6.5	1.5	33.6	2.8	6.2	2.7	4.5	35.1	203.3	48.4	6.2	28.1	46.9	46.6	82.1	66.5	20.0	0.9	0.7
Control	NO	NO	6	4.6	14.0	13.0	334.4	10.7	5.5	1.7	29.6	2.4	5.0	2.3	3.9	27.8	197.7	58.2	7.1	23.7	38.1	39.3	65.1	68.3	18.2	1.0	0.5
Control	NO	NO	6	4.8	13.3	12.7	329.1	9.7	5.5	1.4	28.7	2.6	5.0	2.1	3.3	26.4	190.0	58.6	7.1	24.5	39.6	41.5	68.4	69.6	18.7	1.0	0.4
SO2	YES	NO	0	22.3	52.4	48.6	974.8	10.9	6.4	6.1	86.7	7.8	14.0	3.1	13.2	87.6	165.7	81.8	14.9	23.3	42.2	49.1	48.3	70.5	32.8	1.5	1.0
SO2	YES	NO	0	24.0	56.3	48.7	991.0	11.1	7.6	4.8	83.2	6.1	14.5	3.3	12.6	89.7	160.5	94.1	15.4	23.8	37.6	45.4	78.9	64.1	27.7	8.2	1.3
SO2	YES	NO	0	22.5	52.8	47.6	960.1	11.6	7.1	5.2	84.6	6.4	15.0	2.7	12.2	91.2	152.1	81.3	13.0	23.4	40.6	44.1	78.7	69.0	28.0	1.5	1.4
SO2	YES	NO	6	9.3	21.6	20.2	416.7	23.9	7.2	1.7	36.7	3.6	6.1	3.6	4.4	33.8	187.6	83.5	12.0	25.1	35.0	34.5	64.7	68.1	16.8	1.1	0.6
SO2	YES	NO	6	10.2	23.6	21.0	426.4	25.5	7.7	1.9	38.4	3.5	6.5	3.9	4.9	37.3	166.8	92.6	13.3	26.9	46.7	35.8	69.4	74.6	18.3	1.1	1.0
SO2	YES	NO	6	9.4	21.4	20.9	414.7	20.8	6.5	1.5	37.5	4.1	6.8	4.0	4.9	35.7	170.8	92.6	11.2	26.2	42.4	36.3	57.9	73.4	17.8	1.0	1.0
PEF	NO	YES	0	6.5	22.3	16.4	578.2	8.1	3.7	2.4	47.8	4.3	6.9	1.5	6.5	50.4	119.4	45.2	7.4	37.2	32.1	29.0	55.2	47.2	23.6	0.9	0.4
PEF	NO	YES	0	10.0	25.8	17.5	661.8	8.4	3.9	3.2	55.3	4.4	9.8	1.5	8.1	61.5	120.9	62.9	7.8	37.2	37.6	33.1	60.6	48.0	25.7	0.9	0.6
PEF	NO	YES	0	10.3	35.9	16.9	671.2	8.2	3.7	3.0	59.0	4.1	11.1	1.7	8.5	67.6	119.2	46.3	8.1	37.2	36.9	30.0	56.8	47.9	22.9	0.9	0.7
PEF	NO	YES	6	2.8	11.7	9.9	289.7	6.1	2.0	1.2	21.9	1.8	2.1	1.0	1.7	15.6	143.8	44.8	0.5	11.1	28.8	29.7	110.6	58.9	13.4	0.5	0.2
PEF	NO	YES	6	3.0	14.7	10.7	387.9	9.4	2.1	1.7	32.0	2.5	4.2	1.5	3.5	29.1	134.8	46.5	0.5	10.6	33.5	33.4	121.1	50.7	14.3	0.7	0.4
PEF	NO	YES	6	4.0	13.0	11.0	345.5	15.4	1.5	1.6	28.4	2.2	4.1	1.5	3.2	28.9	141.1	46.5	0.6	10.7	13.3	20.9	33.8	52.0	9.5	0.6	0.4
PEF+SO2	YES	YES	0	15.8	53.9	40.4	933.3	9.8	5.4	5.2	81.1	5.3	17.3	2.8	13.8	91.6	155.1	70.4	12.7	30.3	50.6	42.9	63.6	45.8	30.0	1.6	1.1
PEF+SO2	YES	YES	0	19.0	47.1	36.3	887.5	11.3	6.3	4.3	66.3	5.3	14.4	2.3	12.9	84.8	152.9	66.2	12.0	37.2	48.9	37.9	65.2	54.0	30.5	1.4	0.9
PEF+SO2	YES	YES	0	18.2	48.1	35.3	923.8	11.1	5.9	4.2	72.8	6.0	15.8	2.2	13.5	87.6	152.4	67.0	11.6	37.2	47.6	41.3	63.1	51.1	26.4	1.4	1.0
PEF+SO2	YES	YES	6	7.8	18.9	16.5	371.0	27.0	6.0	3.0	29.1	3.1	8.1	2.3	7.2	31.6	175.9	71.2	11.0	15.5	50.0	38.9	55.2	64.2	19.3	1.2	0.7
PEF+SO2	YES	YES	6	7.6	18.3	16.5	352.7	26.9	7.0	1.8	28.2	3.1	6.3	2.7	4.4	31.0	169.4	67.3	10.8	15.3	41.3	35.2	53.7	63.9	15.0	0.9	0.6
PEF+SO2	YES	YES	6	7.9	20.1	16.1	385.1	29.1	5.4	2.0	30.4	2.7	6.0	2.3	4.4	32.2	170.8	67.2	10.5	15.4	43.5	32.3	61.8	63.1	14.9	1.0	0.6

Table S3. Frequency of descriptors reported by the panelists.

Descriptors	Control	SO_2	PEF	PEF+SO_2
Berries	7	5	1	6
Black cherry in alcohol	4	5	4	4
Black pepper	3	3	1	2
Cherry	5	5	1	7
Clove	2	3	2	2
Cut grass	2	1	8	0
Liquorice	4	5	2	5
Plum	5	8	3	6
Raisin	2	4	3	3
Rose	2	1	0	1

格式化表格
